# Leftists and Rightists Differ in Their Cardiovascular Responses to Changing Public Opinion on Migration

**DOI:** 10.1111/psyp.70140

**Published:** 2025-09-04

**Authors:** Feiteng Long, Ruthie Pliskin, Daan Scheepers

**Affiliations:** ^1^ Social, Economic and Organisational Psychology Leiden University Leiden the Netherlands; ^2^ Faculty of Social and Behavioural Sciences Utrecht University Utrecht the Netherlands

**Keywords:** challenge, ideology, migration, public opinion, threat

## Abstract

People may feel stressed when engaging with contentious topics, such as migration. However, when individuals learn that their opinion‐based ingroup is growing or shrinking, they may experience this stress in different ways, namely as a threat or a challenge. In a preregistered study (*N* = 203 Dutch university students), we examined among host society members how progressive and conservative changes (vs. stability) in public opinion on migration interacted with their political ideology to influence cardiovascular reactivity indicative of challenge and threat. Electrocardiography, impedance cardiography, and blood pressure were continuously measured during a one‐ to three‐minute speech task in which participants reflected on the future of interethnic relations in the Netherlands. Additional self‐reported outcomes, including demand and resource appraisals and prejudice towards migrants, were assessed after the speech task. As predicted, progressive change (vs. stability) in public opinion led leftists to exhibit a cardiovascular pattern indicative of relative challenge (relatively lower total peripheral resistance and higher cardiac output) and rightists to display a cardiovascular pattern indicative of relative threat (relatively higher total peripheral resistance and lower cardiac output). Additional analyses suggest that progressive change (vs. stability) increased leftists' resource appraisal regarding the speech and reduced their prejudice towards migrants, while both progressive and conservative changes (vs. stability) increased rightists' prejudice. These findings highlight that a growing opinion‐based ingroup size can function as a resource for coping with the stress of forming and expressing one's opinion on a sensitive societal issue.

## Impact Statement

The present study reveals how changes in public opinion on migration influence the cardiovascular stress responses of ideological leftists and rightists. Compared to stability, progressive changes in public opinion elicit a challenge response (lower total peripheral resistance, higher cardiac output) among leftists, but a threat response (higher total peripheral resistance, lower cardiac output) among rightists. These findings extend the Biopsychosocial Model of Challenge and Threat to political discourse, illuminating how public opinion dynamics shape stress and prejudice during opinion formation and expression.

## 1 Introduction

Over the past decade, public debates on social issues such as migration have become increasingly polarized (Albada et al. 2021; Chinn et al. 2020; Dalton 2021). These debates have influenced recent electoral outcomes around the world, with the rise of anti‐immigrant parties reshaping public opinion and deepening political divides (Völker and Gonzatti 2024). Such contentious societal issues are not strictly abstract or hypothetical. In fact, they shape everyday interpersonal and intergroup encounters, for example, when individuals discuss these topics with friends or family members, engage with media narratives, or deliberate on voting decisions. In these moments, norms reflected in mainstream public opinion can serve as a reference point, which could validate one's own opinions, but could, alternatively, also motivate conformity to the norm when one becomes aware of a distance between the self and the norms of their group. Such processes are motivationally relevant and even stressful, which can be visible at a physiological level (De Wit et al. 2012; Frings et al. 2012; Newton and Sanford 2003; Seery et al. 2016).

In the present research, we employed the biopsychosocial model of challenge and threat (BPS‐CT; Blascovich and Mendes 2010; Blascovich and Tomaka 1996; Seery 2013; for recent reviews, see Behnke and Kaczmarek 2018; Hase et al. 2019, 2025; Jamieson 2017) to examine individuals' cardiovascular responses when presenting their own opinions under different public opinion contexts. Specifically, we examined how progressive and conservative changes in public opinion on migration, compared to stability, influenced individuals' cardiovascular patterns (indicative of challenge and threat) when they reflected on and spoke about future relations between migrants and members of the host society. We particularly focused on how this influence varied depending on individuals' political ideology.

### 1.1 Challenge and Threat When Reflecting on Migration

People may feel stressed when they are required to reflect on polarizing topics such as migration. Under different circumstances, reflecting on migration can result in either a maladaptive cardiovascular stress response, termed “threat,” or a more benign cardiovascular stress reaction, termed “challenge” (Blascovich and Mendes 2010). According to the BPS‐CT (Blascovich and Mendes 2010; Blascovich and Tomaka 1996; Seery 2013), such threat and challenge responses arise during motivated performance situations, that is, *goal‐relevant* situations requiring an *instrumental response* to deal with a certain demand. Moreover, motivated performance situations have a *socially evaluative* character and are in that sense *self‐relevant*, as performance has relevance for one's well‐being. Motivated performance situations occur in many different contexts, such as when taking a test, engaging in athletic performance, or expressing or debating one's opinion on a certain topic.

In psychological terms, challenge and threat states depend on individuals' appraisals of the demands of the motivated performance situation, in combination with the resources available to the person to cope with these demands (Blascovich and Mendes 2010; Blascovich and Tomaka 1996; Tomaka et al. 1993). Situational demands include factors such as the effort required to successfully accomplish the task, but also the uncertainty and potential danger present in the broader context (Blascovich 2008). Resources refer to the skills, knowledge, support, and personal dispositions available to the person to meet these demands (Li et al. 2023; Moore et al. 2014; Trotman et al. 2018). Accordingly, a threat motivational state arises when demands outweigh resources, while a challenge motivational state arises when resources approach or outweigh demands.

In addition to the psychological appraisal processes leading to challenge and threat motivational states, the BPS‐CT also describes the physiological markers of these states. First, a certain level of task engagement, as an indispensable aspect of motivated performance, is marked by activation of the sympathetic nervous system, leading to increased heart rate (HR) and decreased pre‐ejection period (PEP; an index of left ventricular contractile force). That is, under task engagement, which is common for both challenge and threat motivational states, the heart starts beating faster and stronger.

Given task‐engagement, a challenge motivational state is marked by activation of the sympathetic‐adrenomedullary (SAM) axis. In addition to stimulating cardiac activity (increased HR, decreased PEP), SAM activation leads to the release of epinephrine, causing vasodilation and in turn a decrease in total peripheral resistance (TPR; an index of net constriction vs. dilation of the arterials). The combination of increased cardiac activation and decreased vascular resistance leads to an increase in cardiac output (CO), that is, the amount of blood pumped out by the heart, in liters per minute.

Threat, by contrast, is marked not only by SAM activation but also by activation of the hypothalamic–pituitary–adrenal (HPA) axis, which inhibits SAM activation and particularly its vasodilation component. As a result, compared to baseline, TPR remains stable or increases, while CO remains stable or decreases, despite elevated HR and ventricular force (De Wit et al. 2012; Seery 2013). Thus, during motivated performance, a threat state is characterized by lower CO and higher TPR compared to a challenge state (Seery et al. 2010). The cardiovascular indices of challenge and threat have been validated and applied in dozens of studies, providing a new motivational perspective on a variety of topics in psychology, ranging from social facilitation and decision‐making to inter‐ethnic interactions and athletic performance (for reviews, see Blascovich and Mendes 2010; Hase et al. 2019, 2025; Jamieson 2017; Seery 2011).

### 1.2 Reactions to Changes in Public Opinion Among Ideological Leftists and Rightists

When individuals position themselves on a societally polarized topic, with migration an apt example in many contemporary societies, they may be either challenged or threatened (Blascovich et al. 2001; Domen et al. 2022). This may depend, in part, on normative public views on the topic. Indeed, research has shown that public opinion on migration shapes threat perceptions, attitudes, and behavioral intentions towards migrants (De Coninck 2020; Long and Çakmak 2024; Long et al. 2024; Paluck 2009; Wirz et al. 2018). Nonetheless, findings in this literature have been inconsistent regarding how individuals' existing stances on migration, often reflected in their more general political ideologies, condition the way they respond to public opinion (change) on the topic. For example, previous studies suggest that pro‐migrant public opinion or norms can enhance support for migrants, and particularly so among leftists (Igarashi 2022; Long and Çakmak 2024). Conversely, there is also evidence that pro‐migrant public opinion can reduce prejudice most among people who initially hold more negative attitudes towards migrants—often right‑leaning individuals (Long et al. 2024; Oyamot Jr. et al. 2012; Valsecchi et al. 2024).

To shed more light on these seemingly inconsistent findings, it is necessary to look not only at expressed opinions but also at the fundamental motivational processes accompanying such expressions. Moreover, it is also necessary to move beyond self‐reports because the opinions expressed via such measures may be colored by social desirability concerns, which is especially likely when expressing views on socially sensitive topics. This does not mean that self‐reports are useless in these contexts, in particular because they may indeed mimic the attitudes that people express during social interactions. For obtaining a more complete picture, however, it is still crucial to move beyond self‐report measures and incorporate measures that are less susceptible to conscious processes, such as psychophysiological measures. This can illuminate not only explicitly expressed attitudes on a given topic but also the motivational and physiological processes accompanying such expressions.

Under the framework of the BPS‐CT (Blascovich and Mendes 2010; Blascovich and Tomaka 1996; Seery 2013), we propose that changes in public opinion can shape demand and resource perceptions in individuals' situational appraisals when reflecting or speaking on a contentious topic, as a function of their personal stance on the topic. On the one hand, voicing personal opinions on migration is demanding when people perceive their opinion‐based group to be shrinking. In this situation, individuals may perceive a lack of normative support, feel uncertain about their own opinions, and even worry that their positions will be marginalized, neglected, or rejected (i.e., higher demands). As indicated, according to the BPS‐CT, a situation in which demands outweigh resources will lead to a threat motivational state (Blascovich and Mendes 2010; Blascovich and Tomaka 1996; Tomaka et al. 1993). On the other hand, when people think their opinion‐based group is growing larger, this should make them less uncertain about their opinions and feel safer about the status of their opinion‐based group (i.e., lower demands), while simultaneously feeling increased social support (i.e., higher resources). This situation, in which resources outweigh demands, will lead to higher challenge and lower threat (Blascovich and Mendes 2010; Blascovich and Tomaka 1996; Tomaka et al. 1993).

According to this rationale, when one's personal stance runs counter to the direction of change in public opinion, one will perceive their opinion‐based group to be shrinking. The high demands stemming from such a perception will lead to a threat motivational state (Blascovich and Mendes 2010; Blascovich and Tomaka 1996; Tomaka et al. 1993). Hence, when public opinion on migration changes in a progressive direction (as opposed to remaining stable), ideological rightists, who usually hold an anti‐immigrant stance (Cowling et al. 2019; Crawford and Brandt 2020), will show higher threat (lower challenge) when reflecting on their position (Hypothesis 1). In contrast, if individuals hold a stance consistent with the direction in which public opinion changes, they will perceive a growing opinion‐based ingroup and sufficient resources to cope with the task of reflecting and expressing one's opinion on migration. As a result, such a situation, in which resources outweigh demands, will lead to a challenge motivational state (Blascovich and Mendes 2010; Blascovich and Tomaka 1996; Tomaka et al. 1993). Accordingly, we predict that a progressive change in public opinion on migration (compared to stability) will lead to higher challenge (lower threat) among ideological leftists as they reflect on migration (Hypothesis 2).

We only preregistered the influence of progressive change (i.e., Hypotheses 1 and 2) because at the time we launched the study, this was the most prevalent form of social change in the Netherlands, where we collected the data. We were initially interested in the role of such change in the development of negative intergroup attitudes (e.g., Major et al. 2018). However, as explained before, we propose that this is part of a more general phenomenon, namely the threat vs. challenge of a shrinking vs. growing opinion‐based ingroup size. Therefore, a symmetrical relationship may be observed for those on the left and right sides of the political spectrum when facing conservative social changes. We also acknowledge that since we started this research project, political events in many places across the world have greatly increased the relevance of such conservative shifts.

### 1.3 The Present Research

In the present study, we examined how changes in public opinion on migration and political ideology interacted to affect individuals' physiological challenge and threat responses when they reflected on and presented their views regarding the future of interethnic relations. This approach potentially offers new theoretical and empirical insights. First, there is a need in social and political psychological research to complement self‐report measures with implicit measures to circumvent social desirability bias, which is especially relevant in the context of sensitive and polarizing topics, such as migration. That is, physiological measures can offer a unique advantage in uncovering certain states (e.g., threat) that people are reluctant to explicitly talk about (Seery 2013). A second point of innovation concerns the specific context to which we apply the principles of the BPS‐CT, namely societal shifts. While previous work on the BPS‐CT has predominantly focused on small‐scale individual and interpersonal contexts and the more proximal factors leading to threat and challenge in such contexts, the present work focuses on broad societal processes as impetus for physiological challenge and threat responses. In addition to these potential theoretical and empirical contributions, the current work also has practical relevance, for example, in terms of a possible relationship between shifts in public opinion and health outcomes. Previous work has examined the stress that members of minority groups may experience because of their disadvantaged status (Frost and Meyer 2023). Likewise, when a shrinking ideological ingroup size may elicit similar (threat) related processes, this could have important health consequences for members of specific groups in society.

The central hypotheses tested were that progressive change (vs. stability) in public opinion would lead to higher threat (lower challenge) among ideological rightists (Hypothesis 1) and higher challenge (lower threat) among ideological leftists (Hypothesis 2). To test our hypotheses, we adopted a “speech” paradigm in which participants reflected on and presented their opinion on migration after being provided with (contrived) information indicating progressive change, conservative change, or no change (stability) in public opinion on migration, with the stability treatment serving as a control condition. Before and during the speech, we assessed cardiovascular indices of challenge and threat. After that, we measured self‐reported demand and resource appraisals and prejudice towards migrants. As a moderating variable, we assessed individual differences in political ideology.

The materials, data, and code used in the present study are accessible at https://osf.io/gjbkh. The study was preregistered at https://osf.io/2u58v.

## 2 Method

### 2.1 Participants and Sample Size Justification

The sample size was preregistered and determined prior to data collection based on an a priori power analysis conducted using G*Power 3.1 (Faul et al. 2007). We assumed a medium effect size of *f* = 0.25, as observed in Study 2b by Long et al. (2024), which examined the interaction effect of equality norms and social ideology on self‐reported threat of migrants. The analysis suggested a required sample of 158 participants, which would provide an 80% power to detect a minimum effect of *f* = 0.25 in a one‐way ANOVA with three conditions. However, given our preregistered hypotheses, which focused on the interaction between the manipulation and ideology, after data collection we deemed it would be more appropriate to base our sample size on a regression analysis for this interaction rather than on a one‐way ANOVA focusing on the main effect of the manipulation. We therefore conducted a post hoc sensitivity power analysis, which showed that the obtained sample size (see below) provided an 80% power to detect a minimum effect size of *f* ^2^ = 0.07 (i.e., *f* = 0.26) in a linear regression model with five predictors. This effect size is comparable to that reported in other research examining the interaction between social change and individual differences on threat–challenge responses (e.g., Domen et al. 2022).

Anticipating potential exclusions, we recruited 211 Dutch‐speaking undergraduate students, offering either 6.5 euros or course credits as compensation. Data from eight participants were excluded from the final dataset because five of them indicated having a migration background (which was an exclusion criterion), and for the other three, the experiment was terminated at an early stage after they reported feeling unwell. Among the 203 retained cases, two had incomplete self‐report data and one had incomplete cardiovascular data due to technical issues. The 201 participants who completed self‐report measures were aged between 17 and 30 (*M* = 19.33, SD = 1.85), with 173 identifying themselves as women, 29 as men, and 3 as non‐binary.

### 2.2 Procedure

**FIGURE 1:**
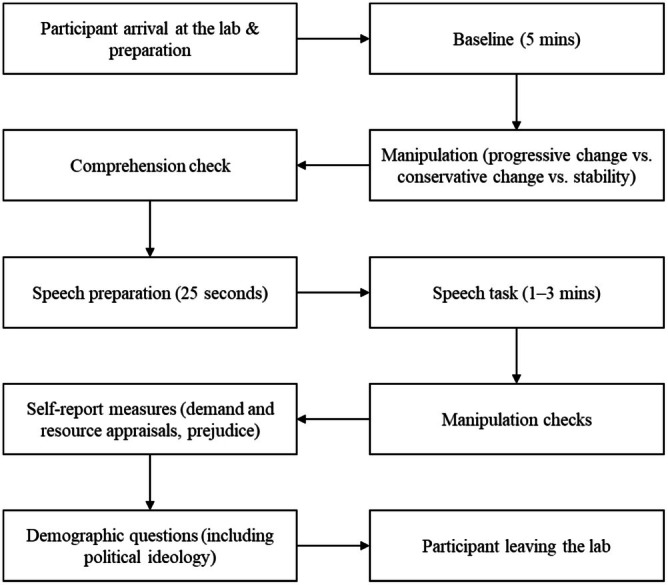
Study procedure.

The entire procedure is illustrated in Figure 1. After arriving at the laboratory, participants were seated individually in a cubicle equipped with a computer, a microphone, and psychophysiological measurement instruments. They were instructed to keep their hand at a specific place at the table and to sit as still as possible throughout the session. Experimental materials were administered in the lab using Qualtrics, a commonly used survey platform. A trained research assistant attached the sensors and electrodes for measuring impedance cardiography (ICG), electrocardiography (ECG) and continuous blood pressure to participants. Then participants' baseline cardiovascular responses were recorded for a five‐minute period, during which they were instructed to sit quietly and relax.

After the baseline recording period, participants read about an ostensibly real survey on Dutch public opinion on migration:

The biannual European Social Survey (ESS) has been tracking attitudes about Migration in various European countries over the past 10 years. A question covered in it is “Do you support major structural changes to strengthen the position of migrants in Dutch society (e.g., removing the majority group‘s privileges, increasing migrants‘ access to positions of power, and encouraging multicultural expressions in schools and workplaces).”

Then, based on random assignment to one of three experimental conditions, participants read about the results of the survey that indicated either progressive change, conservative change, or stability in public opinion on migration. Participants were informed that the number of Dutch people who supported structural changes to strengthen the position of migrants in Dutch society had either increased (progressive change condition), decreased (conservative change condition), or remained unchanged (stability condition) over the past decade, and that this trend was expected to continue in the coming decade. The full manipulation materials can be found in Appendix S1A.

After completing two comprehension checks, participants proceeded to a speech task, where they expressed their opinions on the future of relations between people with and without a migration background in the Netherlands. After 25 s of reflection and preparation^1^, participants delivered the speech in Dutch to the microphone. They were required to speak for at least 1 min and at most 3 min.^2^ During the speech, we recorded participants' voice data and continuous cardiovascular responses. Finally, participants completed manipulation checks, self‐report measures, and demographic questions.

The context (i.e., a speech task) we created fulfills the requirements for a motivated performance situation. First, it was *goal‐oriented*, requiring participants to organize thoughts under time constraints and give an *instrumental response* (i.e., present their views effectively), which necessitated effortful engagement. Moreover, the speech also involved a *socially evaluative* component due to the presence of evaluators (i.e., researchers), which is another key element of motivated performance. Considering that the topic participants spoke about concerns a contentious and polarizing social issue, namely migration, this likely also increased uncertainty about how the speech would be received and the potential implications this may have for social approval, judgment, or rejection. Finally, the lab context and experimenter presence could have further increased participants' focus and motivation.

### 2.3 Checks and Measures

#### 2.3.1 Comprehension and Manipulation Checks

Participants' comprehension of the manipulation information was checked by asking them the name of the national survey mentioned (with choices “Gallup Poll,” “World Values Survey,” “European Social Survey,” and “Opinion Panel”) and the trend in public opinion supporting structural changes in favor of migrants indicated in the text (with choices “increasing,” “decreasing,” “remaining stable,” and “I don't know”). Those who failed either of the two comprehension checks were directed back to the manipulation information and given a second chance to complete the checks again. Ten participants selected incorrect answers once but none did so twice.

In addition, we included three manipulation check items: “To what extent do you think the Dutch majority's views regarding migrants will change?”; “To what extent do you think the Dutch majority will become more positive about migrants?”; “To what extent do you think the Dutch majority will become more negative about migrants? (reversely coded)” Responses were made on 7‐point scales with endpoints 1 (*not at all*) and 7 (*very much*). The first item formed the measure of *perceived change in public opinion* while the other two were averaged to form a measure of *perceived progressive change in public opinion* (*r* = 0.82).

#### 2.3.2 Cardiovascular Measures

To assess challenge and threat motivational states, ICG signals, ECG signals, and blood pressure were continuously measured using a Biopac MP150 system and stored using *Acqknowledge* software (Biopac Systems, Goleta, CA). A sampling rate of 1000 Hz was used. Cardiovascular data were scored using PhysioData Toolbox (Sjak‐Shie 2022). The protocols for physiological data collection and scoring can be found at https://osf.io/k5gjq.

For measuring ICG, the Biopac NICO100c module was used, together with four spot electrodes, two of which were placed at the back of the neck (one at the base of the neck and the other approximately 5 cm higher) and two at the lower back (again separated from each other by approximately 5 cm). The distance between the two inner electrodes was approximately 30 cm. The two outer electrodes injected a small (400 μA) alternating current while the two inner electrodes measured the voltage developed through the thorax volume. As output, the NICO100c provided measures of basal impedance (*Z*_0_) and the rate of change in impedance (*dZ*/*dt*), which, in combination with the ECG, can be used to derive measures of PEP and CO. For determining CO, we first calculated Stroke Volume (SV: the amount of blood pumped out on a single heartbeat), making use of the Kubicek formula (see Sherwood et al. 1990); *Z*_0_ was entered in the formula as a constant, for which we took the mean *Z*_0_ of both baseline and speech task. In turn, CO was calculated by multiplying SV by HR (which was derived from the ECG).

Electrocardiography (ECG) was measured using an ECG100 module and two electrodes: one placed at the suprasternal notch above the top of the sternum, and one at the apex of the heart, on the left lateral margin of the chest approximately at the level of the processus xiphoideus. A ground electrode was not used, as participants were already grounded via the NICO100c.

Blood pressure was measured continuously using a CNAP Monitor 500, which yields a measure of mean arterial pressure (MAP), which, in combination with CO, was used to calculate TPR using the following formula: TPR = (MAP/CO) × 80.

The ECG was used to determine HR and, in combination with the ICG, PEP. A significantly increased HR and decreased PEP (compared to baseline) indicate task engagement, which is a prerequisite for further threat–challenge analysis using CO and TPR (Blascovich et al. 2004; Seery et al. 2010).

Following standard practice, we computed mean scores for HR, PEP, CO, and TPR for the last minute of the baseline and the first minute of the speech task. Then we created reactivity scores by subtracting the baseline scores from the task scores. We Winsorized univariate outliers (defined as 3.3 SDs from the mean) for each reactivity score by recoding them to 1% higher or lower than the adjacent non‐extreme value (Blascovich et al. 2004; Mendes et al. 2002; Scheepers 2017).^3^

Higher CO and lower TPR reactivity scores mark a challenge (relative to threat) motivational state. Following the common practice, we calculated a combined Threat–Challenge Index (TCI). Specifically, we standardized CO and TPR reactivity into *Z*‐scores, multiplied the TPR *Z*‐score by −1, and summed it with the CO *Z*‐score (Blascovich et al. 2004; Seery et al. 2010). Higher scores on TCI represent a greater challenge motivational state, while lower scores indicate a greater threat motivational state.

#### 2.3.3 Self‐Report Measures

Two items assessed participants' *demand and resource appraisals* during the speech task on scales with endpoints 1 (*not at all*) and 7 (*very much*): “How stressful was the speech task you just completed?” (demand appraisal; Tomaka et al. 1993); “How much knowledge did you possess that enabled you to complete the speech task?” (resource appraisal; Blascovich and Mendes 2000).

*Prejudice towards migrants* was measured using a feeling thermometer (0 = *very cold*, 50 = *neutral*, 100 = *very warm*): “In general, on the following scale, how coldly or warmly do you feel towards migrants?” This measure captures affectively driven responses, aligning with the definition of prejudice as “negative affect associated with out‐groups” (Stephan et al. 1999; see also Stephan and Stephan 1993). Scores were reversely coded such that higher values reflect higher prejudice.

**FIGURE 2:**
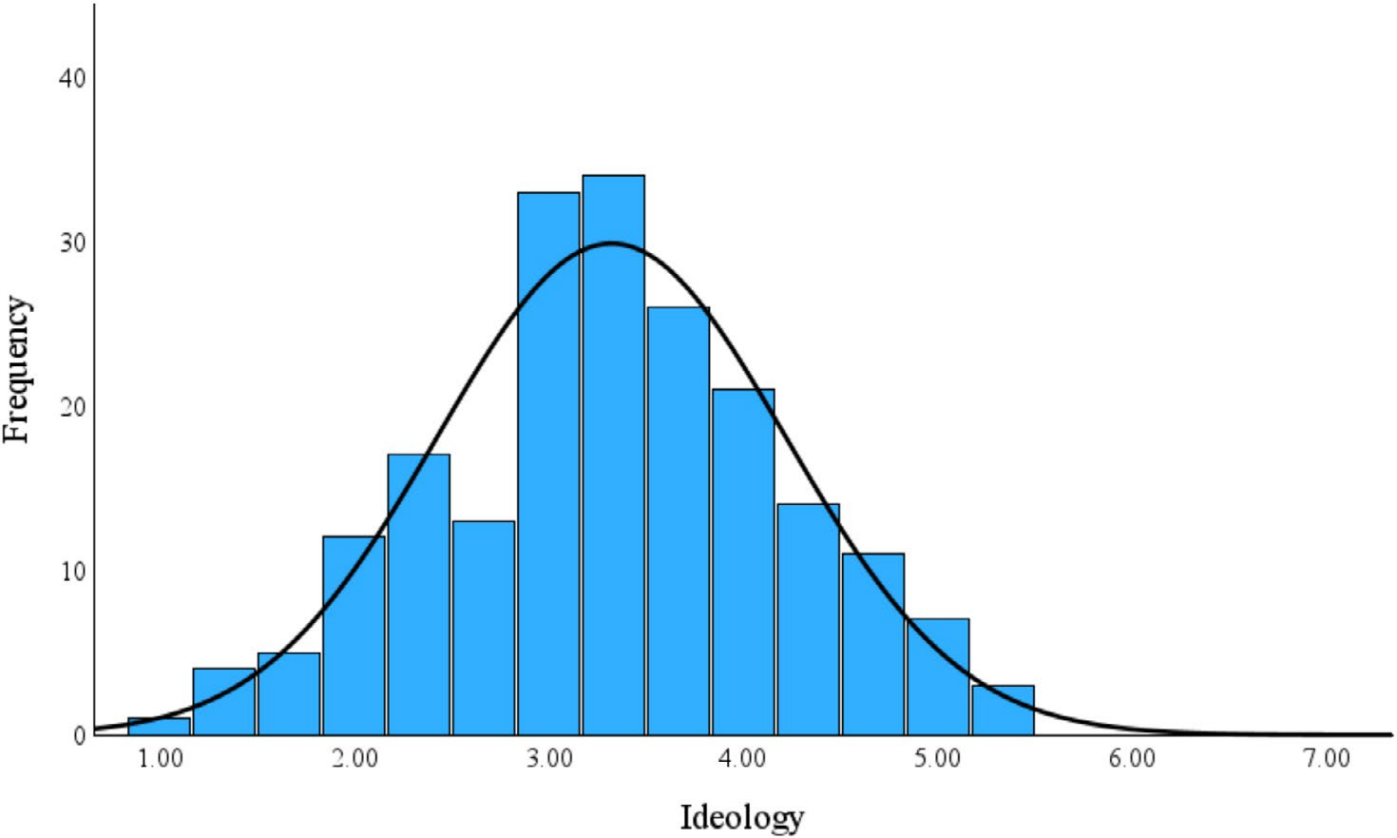
Sample distribution of political ideology.

Three items assessing *political ideology* (α = 0.65) were embedded among the demographic questions (i.e., gender, age, country of birth, parents' country of birth, migration background, and ideology): “On a left‐right spectrum, how would you describe your political orientation? (general ideology; 1 = *very leftist*, 4 = *centrist*, 7 = *very rightist*)”; “How social or liberal are your economic views? (economic ideology; 1 = *very social*, 4 = *neither social nor liberal*, 7 = *very liberal*)”; “How progressive or conservative are your views on social issues? (social ideology; 1 = *very progressive*, 4 = *neither progressive nor conservative*, 7 = *very conservative*).” Scores for the three items were averaged to create a composite ideology score.^4^ The sample was somewhat left‐leaning (*M* = 3.32, SD = 0.90; see Figure 2). Therefore, in analyses for the moderating role of ideology, we explored the simple slope effects at the moderator levels *M −* 1SD, *M* + 1SD, and *M* + 2SD. Our conclusion about leftists and rightists would be based on the simple slope effects at the moderator levels *M* − 1SD and *M* + 2SD.

For exploratory purposes, we also measured distal demands, distal resources, perceived threats, social distance, emotions, right‐wing authoritarianism, social dominance orientation, and group identification (see Appendix S1A for details); however, these variables were not included in the analyses. Additionally, participants' voice data, collected during their speeches, was not analyzed.

### 2.4 Statistical Analysis

Before conducting the main analyses, we performed several preliminary checks. First, we performed one‐way ANOVAs on the two manipulation checks to assess whether the manipulation successfully influenced participants' perceived change in public opinion and perceived progressive change in public opinion.

We then conducted one‐way ANOVAs to examine baseline differences in HR, PEP, CO, and TPR between conditions. Since challenge and threat analyses typically rely on reactivity scores (i.e., task scores minus baseline scores), confirming the absence of between‐condition differences in baseline scores ensures that any observed manipulation effects on threat–challenge indicators are not driven by such pre‐existing differences. As a check for task engagement (which is a prerequisite for further analyses in terms of challenge and threat), we conducted one‐sample *t*‐tests to examine whether HR reactivity was greater than 0 bpm and PEP reactivity was smaller than 0 ms (i.e., baseline levels).

To analyze the main effect of the manipulation on TCI, we performed a one‐way ANOVA. We then tested the core hypotheses regarding the moderating role of political ideology using regression analysis, including the manipulation, ideology, and their interaction as predictors in the model. Additionally, identical ANOVAs and regression analyses were performed with self‐report measures as the dependent variables.

Finally, we explored correlations among continuous variables, including TCI, CO, TPR, demand appraisal, resource appraisal, prejudice towards migrants, and political ideology.

## 3 Results

### 3.1 Manipulation Checks

A one‐way ANOVA revealed that the manipulation of changes in public opinion on migration significantly affected perceived change in public opinion, *F*(2, 198) = 12.37, *p* < 0.001, $\eta_{p}^{2}$ = 0.11. Pairwise comparisons showed that perceived change in public opinion was higher in the progressive‐change condition (*M* = 4.80, SE = 0.13) than in both the stability condition (*M* = 3.88, SE = 0.14), *t*(198) = 4.76, *p* < 0.001, *d* = 0.82, and the conservative‐change condition (*M* = 4.11, SE = 0.14), *t*(198) = 3.58, *p* < 0.001, *d* = 0.61. Interestingly, although participants in the conservative‐change condition indicated society changing to a stronger extent than did those in the stability condition, this difference did not reach significance, *t*(198) = 1.17, *p* = 0.245, *d* = 0.20.

Additionally, the manipulation also significantly affected perceived *progressive* change in public opinion, *F*(2, 198) = 43.72, *p* < 0.001, $\eta_{p}^{2}$ = 0.31. Pairwise comparisons showed that perceived progressive change in public opinion was, as expected, higher in the progressive‐change condition (*M* = 4.76, SE = 0.13) than in both the stability condition (*M* = 4.05, SE = 0.13), *t*(198) = 3.86, *p* < 0.001, *d* = 0.66, and the conservative‐change condition (*M* = 3.05, SE = 0.13), *t*(198) = 9.32, *p* < 0.001, *d* = 1.60. Moreover, perceived progressive change in public opinion was lower in the conservative‐change condition than in the stability condition, *t*(198) = 5.40, *p* < 0.001, *d* = 0.94. We conclude that the manipulation was generally successful.

### 3.2 Cardiovascular Measures

First, we checked if mean scores for HR, PEP, CO, and TPR for the last minute of the baseline varied between conditions. As expected, we found no significant between‐condition differences in baseline HR, *F*(2, 199) = 0.04, *p* = 0.962, $\eta_{p}^{2}$ = 0.00, PEP, *F*(2, 199) = 0.95, *p* = 0.390, $\eta_{p}^{2}$ = 0.01, CO, *F*(2, 199) = 0.01, *p* = 0.991, $\eta_{p}^{2}$ = 0.00, or TPR, *F*(2, 199) = 0.23, *p* = 0.798, $\eta_{p}^{2}$ = 0.00. Further analyses were therefore conducted on reactivity scores (i.e., speech task scores − baseline scores).

#### 3.2.1 Task Engagement

Then, a test for task engagement showed that HR reactivity (*M* = 15.19 bpm, SD = 9.96 bpm) was significantly greater than zero (i.e., baseline), *t*(201) = 21.67, *p* < 0.001, *d* = 1.52, and PEP reactivity (*M* = −11.67 ms, SD = 13.59 ms) was significantly smaller than zero, *t*(201) = 12.20, *p* < 0.001, *d* = 0.86, showing sufficient overall task engagement and goal relevance.

#### 3.2.2 Threat–Challenge Index

Following the preregistered protocol, we first examined the main effect of the manipulation of changes in public opinion on TCI using one‐way ANOVA, although we did not have a specific hypothesis for it. As preregistered, we further tested the core hypotheses focused on the moderating role of political ideology using regression analysis, including the manipulation, ideology, and their interaction as predictors in the model. The manipulation of changes in public opinion was dummy‐coded with the stability condition as the reference category.^5^

**FIGURE 3:**
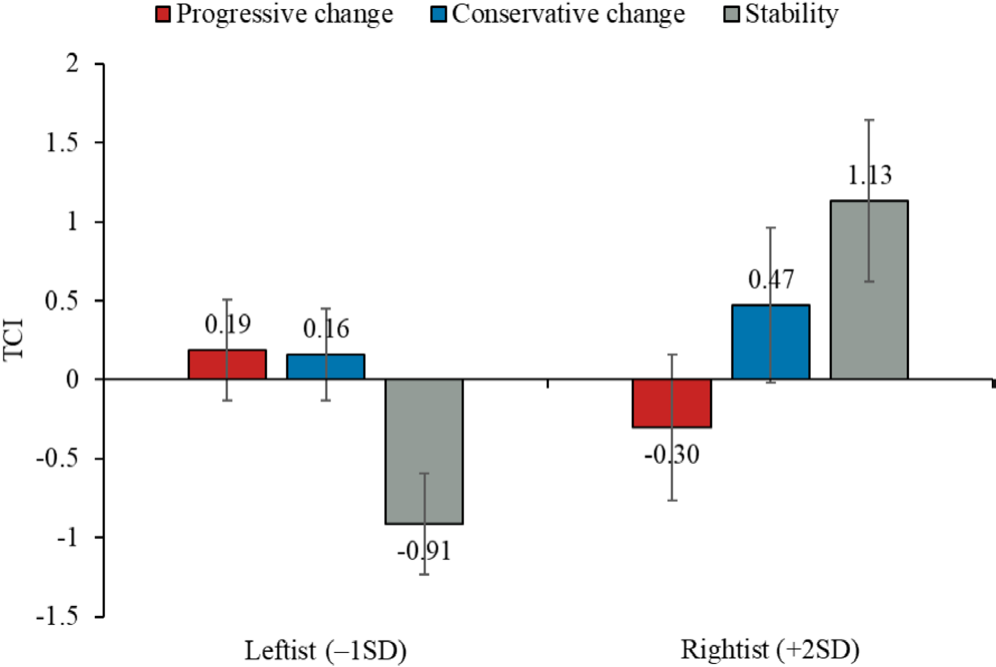
Effect of the manipulation on Threat–Challenge Index (TCI), as moderated by political ideology.

The manipulation of changes in public opinion showed no significant effect on TCI, *F*(2, 199) = 1.20, *p* = 0.302, $\eta_{p}^{2}$ = 0.01. However, the regression analysis provided evidence for both Hypotheses 1 and 2. That is, there was a significant interaction between progressive change (vs. stability) and ideology (see Figure 3), *b =* −0.95, SE = 0.36, *t*(194) = −2.66, *p* = 0.009, 95% CI [−1.65, −0.24]. As predicted, a progressive change in public opinion (vs. stability) led to higher challenge (lower threat) for ideological leftists (–1SD: *b =* 1.10, SE = 0.45, *t*(194) = 2.45, *p* = 0.015, 95% CI [0.22, 1.99]) and higher threat (lower challenge) for ideological rightists (+2SD: *b =* −1.43, SE = 0.69, *t*(194) = −2.06, *p* = 0.041, 95% CI [−2.80, −0.06]; +1SD: *b =* −0.58, SE = 0.43, *t*(194) = −1.35, *p* = 0.178, 95% CI [−1.43, 0.27]). The interaction effect of conservative change (vs. stability) and ideology on TCI was not significant, *b =* −0.65, SE = 0.35, *t*(194) = −1.86, *p* = 0.064, 95% CI [−1.34, 0.04].

In addition, we conducted parallel analyses on the individual components of TCI, namely CO and TPR. The results can be found in Appendix S1E.

### 3.3 Self‐Report Measures

To test the main and interaction effects of the manipulation and political ideology on each self‐report measure (i.e., resource appraisal, demand appraisal, and prejudice towards migrants), we performed the same one‐way ANOVA and regression analysis as for TCI.

**FIGURE 4:**
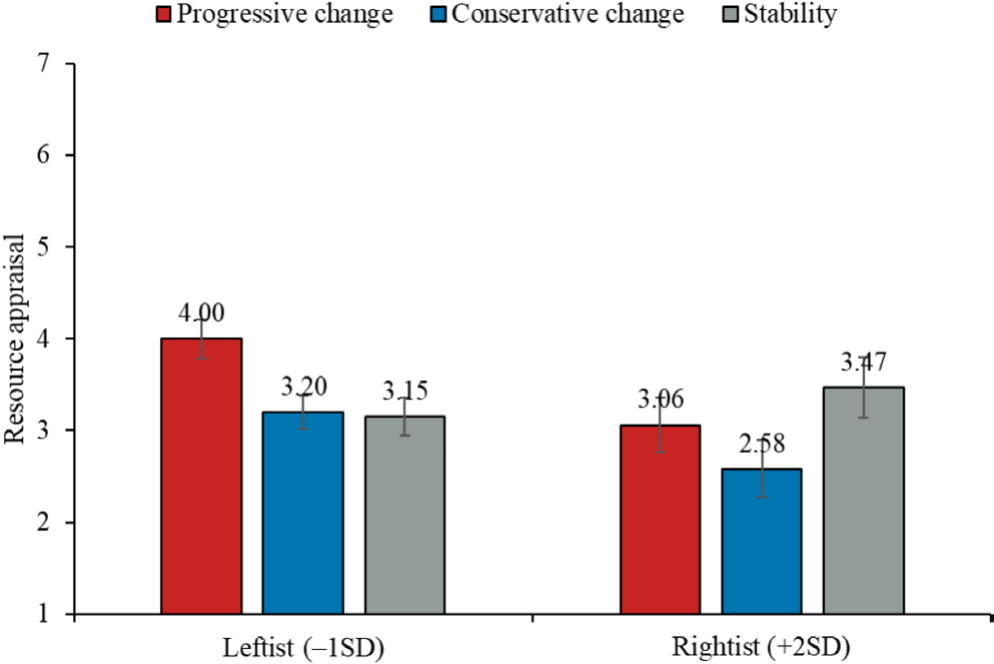
Effect of the manipulation on resource appraisal, as moderated by political ideology.

The ANOVA suggested that the manipulation effect on resource appraisal was significant, *F*(2, 198) = 5.26, *p* = 0.006, $\eta_{p}^{2}$ = 0.05. Pairwise comparisons showed that participants in the progressive‐change condition (*M* = 3.65, SE = 0.14) perceived higher resources to cope with the speech task than those in the stability condition (*M* = 3.26, SE = 0.14), *t*(198) = 1.99, *p* = 0.048, *d* = 0.34, and those in the conservative‐change condition (*M* = 3.02, SE = 0.14), *t*(198) = 3.21, *p* = 0.002, *d* = 0.55. There was no significant difference in resource appraisal between the conservative‐change and stability conditions, *t*(198) = 1.21, *p* = 0.229, *d* = 0.21. To test the moderating role of political ideology, we performed further regression analysis as we did for TCI. It revealed a significant interaction between progressive change (vs. stability) and ideology on resource appraisal, *b =* −0.47, SE = 0.23, *t*(195) = −2.03, *p* = 0.043, 95% CI [−0.92, −0.01], while the interaction between conservative change (vs. stability) and ideology was not significant, *b =* −0.35, SE = 0.22, *t*(195) = −1.57, *p* = 0.119, 95% CI [−0.79, 0.09]. As shown in Figure 4, a progressive change (vs. stability) in public opinion increased ideological leftists' resource appraisal (–1SD: *b =* 0.85, SE = 0.29, *t*(195) = 2.90, *p* = 0.004, 95% CI [0.27, 1.43]), but did not have a significant influence among ideological rightists (+2SD: *b =* −0.41, SE = 0.45, *t*(195) = −0.92, *p* = 0.361, 95% CI [−1.29, 0.47]; +1SD: *b =* 0.01, SE = 0.28, *t*(195) = 0.04, *p* = 0.971, 95% CI [−0.54, 0.56]). As our sample was somewhat left‐leaning, this may have explained the significant main effect of progressive change (vs. stability) on perceived resources.

Parallel analyses on demand appraisal indicated no significant main effect of the manipulation, *F*(2, 198) = 0.09, *p* = 0.915, $\eta_{p}^{2}$ = 0.00. Additionally, neither the interaction effect of progressive change (vs. stability) and ideology, *b =* −0.15, SE = 0.28, *t*(195) = −0.55, *p* = 0.580, 95% CI [−0.70, 0.39], nor that of conservative change (vs. stability) and ideology, *b =* −0.20, SE = 0.27, *t*(195) = −0.74, *p* = 0.462, 95% CI [−0.73, 0.33], reached significance.

**FIGURE 5:**
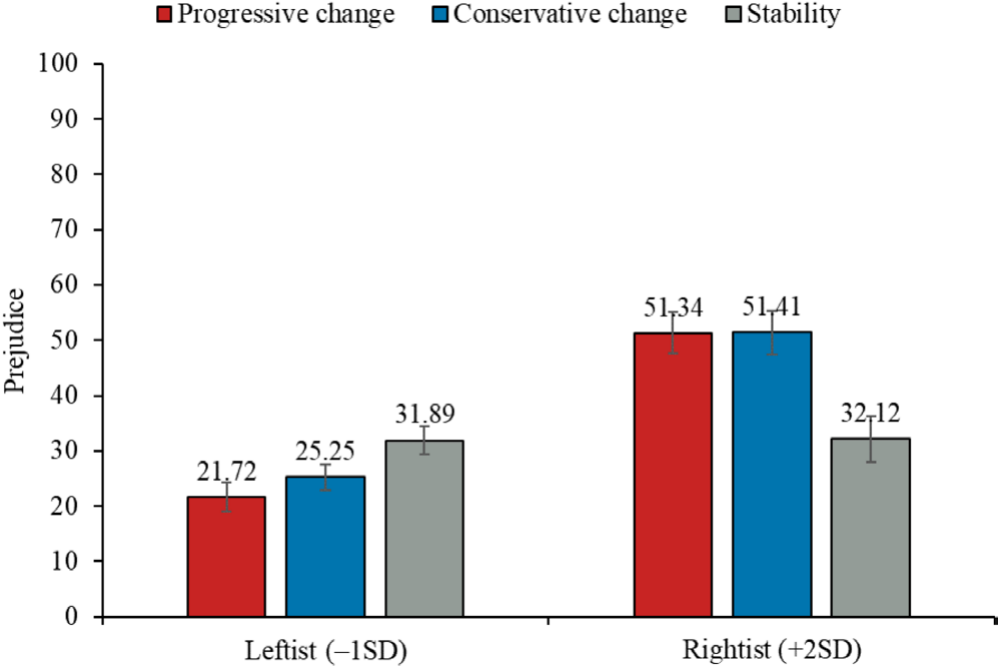
Effect of the manipulation on prejudice, as moderated by political ideology.

The same test for prejudice towards migrants as the outcome variable demonstrated no significant main effect of the manipulation, *F*(2, 198) = 0.06, *p* = 0.938, $\eta_{p}^{2}$ = 0.00. Nonetheless, the interaction between progressive change (vs. stability) and ideology, *b =* 10.94, SE = 2.93, *t*(195) = 3.74, *p* < 0.001, 95% CI [5.17, 16.71], and that between conservative change (vs. stability) and ideology, *b =* 9.65, SE = 2.83, *t*(195) = 3.41, *p* < 0.001, 95% CI [4.06, 15.24], were both significant. The interactions are shown in Figure 5: a progressive change (vs. stability) in public opinion decreased ideological leftists' (–1SD: *b =* −10.17, SE = 3.72, *t*(195) = −2.73, *p* = 0.007, 95% CI [−17.51, −2.84])—but increased ideological rightists' (+2SD: *b =* 19.22, SE = 5.68, *t*(195) = 3.38, *p* < 0.001, 95% CI [8.01, 30.42]; +1SD: *b =* 9.42, SE = 3.52, *t*(195) = 2.68, *p* = 0.008, 95% CI [2.48, 16.37])—prejudice towards migrants. Moreover, a conservative change (vs. stability) in public opinion increased ideological rightists' prejudice towards migrants (+2SD: *b =* 19.28, SE = 5.78, *t*(195) = 3.34, *p* = 0.001, 95% CI [7.89, 30.68]; +1SD: *b =* 10.64, SE = 3.67, *t*(195) = 2.90, *p* = 0.004, 95% CI [3.41, 17.88]), but did not have a significant influence among ideological leftists (–1SD: *b =* −6.64, SE = 3.49, *t*(195) = −1.90, *p* = 0.059, 95% CI [−13.52, 0.25]).

### 3.4 Correlations

Finally, we examined the correlations between cardiovascular responses and self‐report measures (see Table F1 in Appendix S1F). The most noteworthy correlation was between demand appraisal and a cardiovascular response pattern of challenge (i.e., demand appraisal's positive associations with TCI and CO, and a negative association with TPR).^6^ Moreover, the cardiovascular measures did not correlate with resource appraisal, prejudice towards migrants, or political ideology. Among the self‐report measures, prejudice towards migrants was associated with higher demand appraisal, lower resource appraisal, and a more conservative ideology.

## 4 Discussion

In the present study, we tested two hypotheses concerning how progressive change in public opinion on migration influences threat–challenge responses among ideological rightists and leftists when reflecting on and talking about migration. The findings supported our hypotheses, showing that progressive change (vs. stability) led to a cardiovascular pattern indicative of higher challenge (lower threat) among ideological leftists and a pattern indicative of higher threat (lower challenge) among ideological rightists. Additionally, we found that progressive change (vs. stability) in public opinion increased ideological leftists' self‐reported resource appraisal in the speech task and reduced their self‐reported prejudice towards migrants. Finally, both progressive and conservative changes (vs. stability) increased ideological rightists' prejudice towards migrants.

### 4.1 Implications

The present research has shown how broad societal shifts can impact individual physiological responses as a function of political ideology. In this sense, the current work extends the BPS‐CT, which typically focuses on individual and interpersonal contexts, by examining broader societal tendencies as a source of challenge and threat. When one's opinion‐based group grows, one may perceive this as increased support and exhibit a challenge motivational state. In contrast, when learning that one's ingroup size is shrinking, this is likely to lower perceived support, in turn leading to a threat motivational state.

The present results align with previous research indicating that individuals seek support for their stances, which affirms their correctness. This, in turn, can enhance self‐esteem and social status while reinforcing a sense of belonging (Ballara 2023; Hillman et al. 2023). Conversely, a lack of support threatens belonging, triggering aversive avoidance and increasing defensiveness (Hillman et al. 2023; Seery et al. 2016). In this sense, the present work demonstrates that societal shifts can function either as a resource or a source of vulnerability depending on one's political ideology, thereby shaping physiological challenge and threat responses.

More broadly and related to the point presented above, our study contributes to the literature examining reactions to changes in group status and power. According to social identity theory (Tajfel and Turner 1979), individuals categorize themselves and others into social groups (e.g., ideological leftists or rightists) and positively evaluate their ingroup relative to the outgroup to maintain a positive social identity. Perceived declines in ingroup status and power threaten this social identity, leading to defensive reactions (Aquino and Douglas 2003; Scheepers et al. 2009; Steele et al. 2002). Conversely, when one perceives an improved ingroup status and power, this strengthens their positive identity and helps restore a sense of control and agency to address challenges and uncertainties (Cohen and Garcia 2008). Our findings align with these theories, showing that individuals displayed a cardiovascular pattern indicative of threat when their ideological groups appeared to be losing influence (i.e., public opinion shifted against their ingroup values). In contrast, public opinion reflecting the values of one's ideological group provided validation, leading to a cardiovascular pattern indicative of challenge. In this sense, our findings are in keeping with the social identity perspective, illustrating the threat of changing power relations in the context of political ideologies.

In addition to the main findings discussed above, an interesting pattern emerged: rightists in both progressive change and conservative change conditions expressed higher prejudice towards migrants compared to the stability condition. Different processes may underlie these increases in prejudice. In the conservative change condition, heightened prejudice among rightists may reflect greater confidence in expressing the normative ingroup opinion when this view is on the rise. The lower prejudice observed among leftists in the progressive change condition (compared to the control) is also in line with this reasoning. By contrast, the progressive change condition (vs. control) elicited relative threat among rightists, and their increased prejudice may be interpreted as a defensive response to unwanted social change (Riek et al. 2006). Although the cardiovascular responses are broadly consistent with these explanations, the absence of a direct relation between cardiovascular responses and prejudice makes it premature to draw a definitive conclusion about the mechanisms driving prejudice.

Finally, and from a more practical perspective, our findings are relevant to the potential downstream implications for health and psychological resilience. Previous research has suggested that a persistent lack of support for one's core beliefs leads to chronic stress and anxiety (Pascoe and Smart Richman 2009). Minority group members may be particularly prone to such detrimental consequences—when their views are marginalized or stigmatized, the resulting sense of lacking support can foster emotional exhaustion and social withdrawal (Frost and Meyer 2023; Jost and Banaji 1994; Major and O'Brien 2005). Our findings indicate that not only current minority status, but also the prospect of becoming a minority (e.g., shrinking group‐size), elicits maladaptive cardiovascular responses, which may, in the long run, undermine mental and physical health (Sapolsky 2005; Scheepers and Ellemers 2019).

### 4.2 Limitations

Despite the contribution of this study, several limitations should also be acknowledged. One notable limitation concerns the composition of our sample, which consisted of Dutch‐speaking undergraduate students leaning, on average, towards the left side of the ideological spectrum. This limited diversity may restrict the generalizability of our findings to broader populations and particularly to ideological rightists. Addressing this issue in future research would require recruiting a more diverse and ideologically balanced sample.

Relatedly, a second issue concerns the generalizability of our findings across different societies and cultural contexts. While migration is a widely discussed topic globally, the term “migrant” may evoke different associations depending on the societal context. For example, in the Netherlands, the most salient migrant groups migrate from the Middle East, North Africa, and Eastern Europe, and in the United States, migration from Central America is more salient (Jennissen et al. 2023; Power 1979; Sidler et al. 2024). Attitudes towards these migrant groups can be shaped by different factors (e.g., religion or economy). Nonetheless, despite variations in specific migrant groups and in underlying motives behind attitudes towards these groups, the fundamental psychological processes driving individuals' responses to shifting public opinion are likely to be generic. That is, we propose that the central process we examine here relates to a more general growth or decline in support for one's attitudes and may not necessarily be tied to the specific topic examined in this study (i.e., migration).

Additionally, the existing normative background of a society, and especially whether it is actually shifting to be more progressive or conservative, plays a crucial role in how individuals process and interpret information about changes in public opinion. In fact, whether Dutch public opinion is becoming more progressive remains somewhat ambiguous and open to interpretation. On one hand, Ruisch et al. (2021) found across various cultures (including the Netherlands), and in over 20 studies, that both liberals and conservatives perceived that the world had become more progressive over the last 50 years. However, it is debatable whether this trend (or its perception) has continued and will continue in the coming years. During the period in which we conducted our study (between February 2023 and February 2024), public opinion on migration in the Netherlands was shifting from a more open stance towards migration to increased opposition against it, as also evidenced by the rise of the right‐wing populist Party for Freedom (De Jonge and Voerman 2025). Despite this, during this ambivalent period, there were signs of both progressive and conservative changes, allowing for a sufficiently convincing case to be made for either the progressive or conservative change message presented to the participants. As such, it may have been a uniquely fitting opportunity to convincingly manipulate the direction in which norms were trending. Nonetheless, it remains a question whether the patterns observed at the unique moment of data collection, particularly that rightists were threatened by progressive change while leftists did not have a comparable threat response to conservative change, would replicate in the current sociopolitical climate, in which public opinion has been shifting rightwards in multiple democracies. Such a real‐world public opinion shift could lead the experimental condition of conservative change to be more credible and thus more threatening.

We also note that our manipulation of conservative change did not significantly increase perceived change in public opinion compared to the stability condition. This may help explain why we observed that progressive change influenced left‐ and right‐leaning participants' threat–challenge responses, whereas conservative change did not show corresponding effects. One possible explanation for the non‐significant manipulation effect of conservative change (vs. stability) on perceived change in public opinion is that the common understanding of social change typically refers to forward progress—such as dismantling privileges, increasing equality, and enhancing inclusion—rather than change towards greater inequality (Kay and Friesen 2011). Instead, conservative change may be interpreted as a reinforcement of the status quo, as it seeks to reverse social trends and reinstall old systems (Eidelman and Crandall 2012; Proch et al. 2019). This, in turn, may lead individuals to anchor conservative change closer to stability in their perceptions. Thus, in addition to securing samples that are more ideologically balanced, future research should also explore whether participants' implicit assumptions about societal change skew towards progress.

In addition, the present data provide limited insight into the behavioral consequences of challenge and threat in the context of public opinion shifts. We found only limited evidence for a relationship between psychophysiological and self‐report measures (see Table F1 in Appendix S1F). For example, although we observed a consistent pattern among ideological leftists that a progressive change in public opinion increased physiological challenge and resource appraisal during the speech task and reduced their prejudice towards migrants, the threat–challenge index did not significantly correlate with self‐reported resource appraisal or prejudice. This lack of observed correlations, however, is theoretically and methodologically understandable. Theoretically, motivational processes can be subtle and unconscious (Nisbett and Wilson 1977; Seery 2013), meaning that individuals may not be able to accurately self‐report their immediate reactions. From a methodological perspective, the absence of clear and consistent relationships between physiological and psychological measures may reflect the fact that the two types of data have different psychometric properties and were collected at different time points (see Trotman et al. 2018 for the importance of timing in this context). Together, these theoretical and methodological factors may have reduced the likelihood of detecting associations between psychophysiological and self‐report measures.

A further limitation of the present study concerns the operationalization of demands and resources using self‐report items. With the selection of items we included, we sought to cover demands and resources at both proximal and distal levels. The proximal measures are related to the direct speech situation—the stress it causes and the knowledge one has to perform well in the speech task. In contrast, the distal measures capture more abstract psychological concepts (e.g., “the threat of migration”) that may still feed into the evaluation of the concrete speech situation as a source of uncertainty, or as an “affective cue” (Blascovich and Mendes 2000; see Appendix S1A for all items included). In the present study, we found evidence only for the role of (in)consistency between personal belief and public opinion change in shaping one of the proximal appraisal components, namely the resource of “knowledge to complete the task.” However, we did not observe any effect on the proximal demand item (“how stressful was the task?”; see Tomaka et al. 1993) or on distal appraisal items on “the threat of migration”.

One reason for the modest effects on self‐reported demand and resource evaluation may be that people lack conscious access to these evaluations or may be unwilling to report them openly (Blascovich and Mendes 2000; Weisbuch‐Remington et al. 2005). This is, of course, also a primary reason for assessing challenge and threat using more implicit, physiological measures. Indeed, a more comprehensive understanding of the psychology of challenge and threat is ultimately achieved by triangulating their subjective, physiological, and behavioral aspects. However, it should be noted that there are also additional methodological limitations in our approach to measuring demands and resources, one of them being the retrospective nature of these measures (Trotman et al. 2018). Although measuring demands and resources just before task completion has potential drawbacks (e.g., it may distract participants, and reflections on or responses to the items may downregulate the experienced threat), an important advantage is that it captures demands and resources “in the moment,” similar to the physiological measures—something the timing of our measurement precluded. In future research, we believe that the conceptualization and operationalization of demands and resources, including the distinction between distal and proximate components, could benefit from further systematic development and validation (Moore et al. 2014). Moreover, a more consistent approach to assessing demands and resources is warranted to facilitate cross‐study comparisons and meta‐analytic efforts (Hase et al. 2019).

Finally, our measure of prejudice towards migrants focused on its general affective component, i.e., overall felt positivity (warmth) towards migrants, and did not capture other, more nuanced forms of prejudice that may be reflected in, for example, discrete emotions felt towards specific outgroups. Future research may consider adopting multiple operationalizations of prejudice towards migrants, such as social distance (Halperin et al. 2007), political intolerance (Freitag and Rapp 2015), and negative emotions (Fiske et al. 2002), to capture other important components.

### 4.3 Conclusion

The present study demonstrates that shifts in public opinion on migration interact with individuals' political ideology to shape cardiovascular responses when expressing their views on the topic. These findings highlight the role of alignment between public opinion trends and personal beliefs as a psychological resource for managing the stress associated with, for instance, participating in political discourse. By shedding light on the dynamic interplay between public opinion and individual ideology, this work advances the broader literature on stress, coping, and changes in group status and power. In doing so, it also offers potential insights into reducing threat responses and fostering more constructive dialogue around polarizing issues.

## Author Contributions

**Feiteng Long:** conceptualization, data curation, formal analysis, funding acquisition, investigation, methodology, project administration, resources, software, validation, visualization, writing – original draft, writing – review and editing. **Ruthie Pliskin:** conceptualization, methodology, resources, supervision, writing – review and editing. **Daan Scheepers:** conceptualization, methodology, resources, supervision, validation, writing – review and editing.

## Ethics Statement

This research adheres to the ethical guidelines specified in the APA Code of Conduct as well as the Netherlands Code of Conduct for Research Integrity, and was reviewed and approved by the Psychology Research Ethics Committee of Leiden University under reference number [2022‐12‐11‐D.T. Scheepers‐V1‐4388].

## Conflicts of Interest

The authors declare no conflicts of interest.

## Supporting information


**Data S1:** psyp70140‐sup‐0001‐DataS1.docx.

## Data Availability

The data that support the findings of this study are openly available in OSF at http://doi.org/10.17605/osf.io/gjbkh.

## Endnotes

^1^
The procedure aimed to mimic real‐life political discussions, for example, with friends, colleagues, or others, in which individuals typically have limited time to deliberate on their position and think about how to present it. At the same time, since migration was a broadly discussed topic in the Netherlands at the time of the study, participants had likely already considered the subject and formed opinions on it. Avoiding excessive preparation could prevent overthinking and unnatural responses. We report analyses for cardiovascular data recorded during the period of reflection and preparation for the speech task in Appendix S1B.

^2^
On average, participants spoke for 93.55 s, with moderate variability (SD = 29.63).

^3^
Three reactivity scores for HR, one for PEP, two for CO, and five for TPR were Winsorized.

^4^
The Cronbach's α for the three‐item composite was just acceptable. General ideology was strongly associated with economic ideology (*r* = 0.46, *p* < 0.001) and social ideology (*r* = 0.52, *p* < 0.001), while the correlation between economic ideology and social ideology was moderate, though significant (*r* = 0.19, *p* = 0.007). In Appendix S1C, we report the same analyses for cardiovascular measures of threat and challenge as well as self‐report measures as in the main text, using economic ideology and social ideology separately as moderators.

^5^
As reported in Appendix S1D, we conducted the same analyses with speech duration being controlled for.

^6^
Although this correlation may be surprising at first (as challenge is typically more strongly associated with resources), this finding is not entirely at odds with the BPS‐CT. Indeed, challenge is likely to be particularly strong in case both resources and demands are high.
