# Supplementary material for: Leftists and Rightists Differ in Their Cardiovascular Responses to Changing Public Opinion on Migration
Source: Psychophysiology. 2025 Sep 4;62(9):e70140. doi: 10.1111/psyp.70140 (PMC12411664; doi:10.1111/psyp.70140)
Supplement: Supplementary file 1 — Data S1: psyp70140‐sup‐0001‐DataS1.docx. [file PSYP-62-e70140-s001.docx]

**Appendix A. Materials**

**Baseline Measurement**

To begin with, we would like to take a baseline measurement, that is, a measurement while you are sitting still. For this, we ask you to watch the following video, for a few minutes. Stay as calm as possible while you watch it. Also for the remainder of the study we would like to ask you to sit as still as possible, and to avoid making more “large” movements.

**Manipulation**

**<Change Towards Equality>**

The biannual European Social Survey (ESS) has been tracking attitudes about Migration in various European countries over the past 10 years. A question covered in it is “**Do you support** **major structural changes to strengthen the position of migrants in Dutch society** (e.g., removing the majority group’s privileges, increasing migrants’ access to positions of power, and encouraging multicultural expressions in schools and workplaces)”. In the Netherlands, the survey’s results reveal that the percentage of Dutch people who support such changes **has been steadily increasing**, from about 36% in 2012 to about 50% in 2022. In light of this trend, the ESS researchers expect this support for changes to become the **majority** view among Dutch people in the coming years.

As you can see in the graph below, Dutch people’s support for major structural changes to strengthen the position of migrants **has been steadily increasing** in the past decade and is expected to become the **majority** view among Dutch people in the coming years.

***
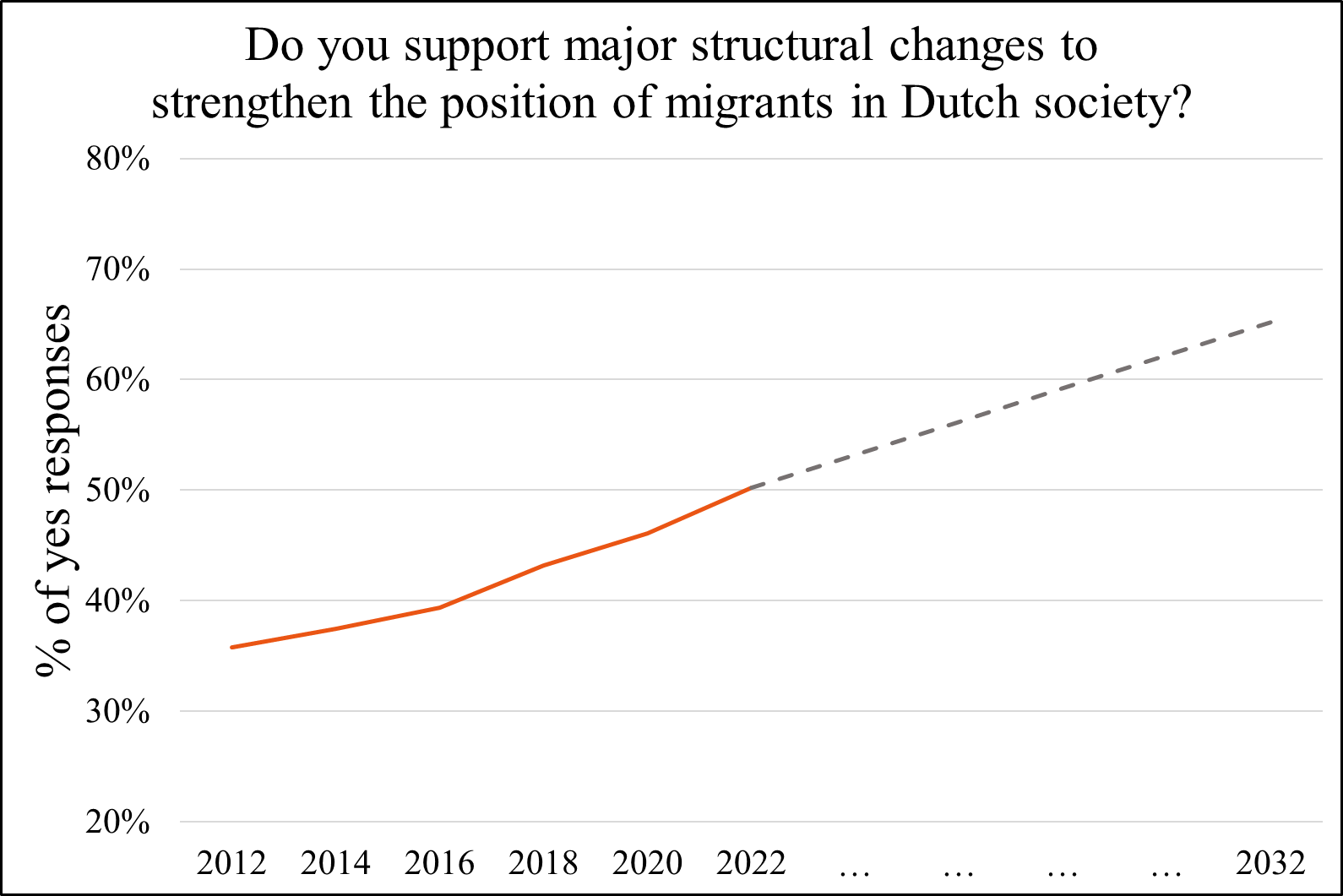
***

In summary, the ESS national survey revealed that support for major structural changes to strengthen the position of migrants is **increasing** and will become the **majority** view in the Netherlands**.**

**********

**<Change Towards Inequality>**

The biannual European Social Survey (ESS) has been tracking attitudes about Migration in various European countries over the past 10 years. A question covered in it is “**Do you support** **major structural changes to strengthen the position of migrants in Dutch society** (e.g., removing the majority group’s privileges, increasing migrants’ access to positions of power, and encouraging multicultural expressions in schools and workplaces)”. In the Netherlands, the survey’s results reveal that the percentage of Dutch people who support such changes **has been steadily decreasing**, from about 64% in 2012 to about 50% in 2022. In light of this trend, the ESS researchers expect this support for changes to become a **marginal** view among Dutch people in the coming years.

As you can see in the graph below, Dutch people’s support for major structural changes to strengthen the position of migrants **has been steadily decreasing** in the past decade and is expected to become a **marginal** view among Dutch people in the coming years.


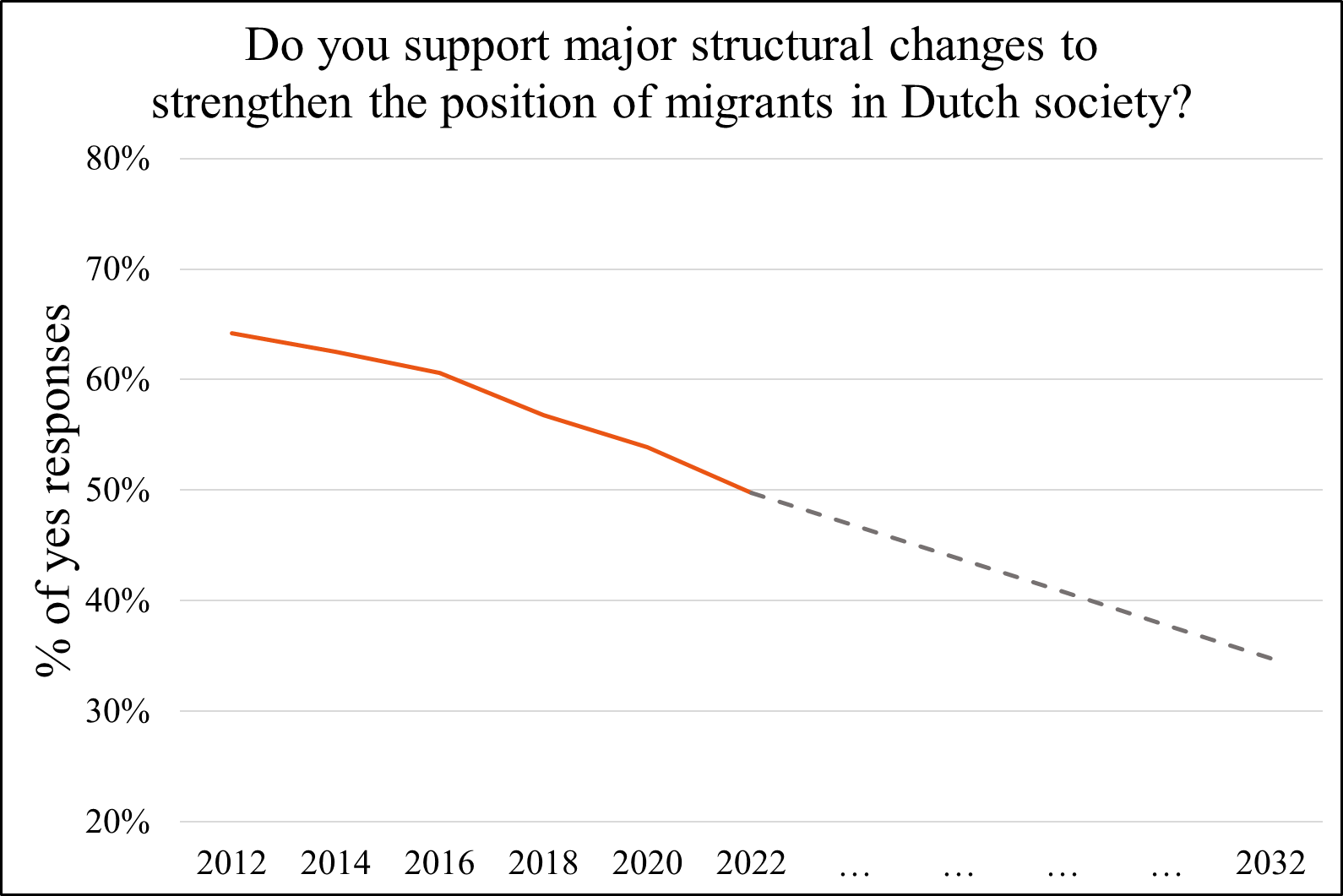


In summary, the ESS national survey revealed that support for major structural changes to strengthen the position of migrants is **decreasing** and will become a **marginal** view in the Netherlands**.**

**********

**<Stable Norm>**

The biannual European Social Survey (ESS) has been tracking attitudes about Migration in various European countries over the past 10 years. A question covered in it is “**Do you support** **major structural changes to strengthen the position of migrants in Dutch society** (e.g., removing the majority group’s privileges, increasing migrants’ access to positions of power, and encouraging multicultural expressions in schools and workplaces)”. In the Netherlands, the survey’s results reveal that the percentage of Dutch people who support such changes **has** **remained stable** over the past decade. In light of these findings, the ESS researchers **do not expect much change** in Dutch support for such measures in the coming years.

As you can see in the graph below, about half of Dutch people support for major structural changes to strengthen the position of migrants and another half do not. This percentage **has remained stable** in the past decade and is expected to **hardly change** in the coming years.

*
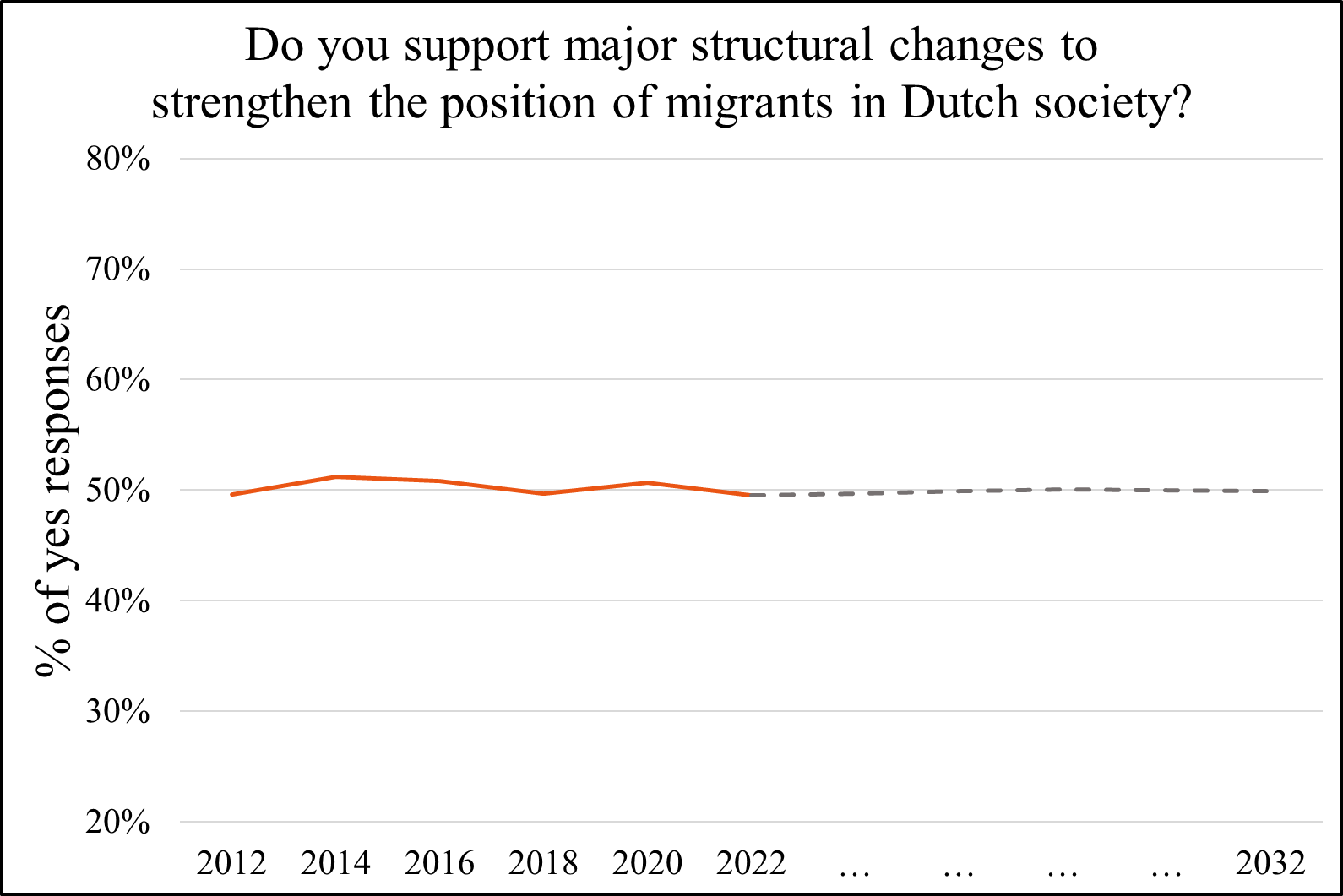
*

In summary, the ESS national survey revealed that about half of Dutch people support major structural changes to strengthen the position of migrants and another half do not. This percentage **remains stable** and will **hardly change** in the coming years**.**

**Speech**

**<Comprehension Checks>**

To ensure you have understood the information correctly, we would like you to answer two questions regarding the information you just read.

What is the name of the national survey mentioned in the text you just read?

(Gallup Poll; World Values Survey; European Social Survey; European Values Study)

According to the national survey, how is the trend in the percentage of Dutch people who support major structural changes to strengthen the position of migrants in Dutch society?

(Increasing; decreasing; remaining stable; I don’t know)

**<Speech Preparation>**

Now we would like to ask you give a short speech (1-3 minutes) expressing your own opinions on the future of relations between native Dutch and migrants in the Netherlands.

You don’t have to prepare for this, you can just talk freely and spontaneously like in a conversation with a friend.

**<Speech>**

Now let’s start to talk about your own opinions on the future of relations between native Dutch and migrants in the Netherlands (at least 1 minute and at most 3 minutes).

You can continue the speech by clicking “Record”. Click it again to end it when you finish the speech.

**Measures**

**<Manipulation Checks>**

To what extent do you think the Dutch majority’s views regarding migrants will change?

To what extent do you think the Dutch majority will become more **positive** about migrants?

To what extent do you think the Dutch majority will become more **negative** about migrants?

(1 = not at all, 7 = very much)

**<Perceived Proximal Demand & Resources>**

Regarding the short speech that you just gave, please answer the following questions.

How stressful was the speech task you just completed? (1 = not stressful at all, 7 = very stressful)

How much knowledge did you possess that enabled you to complete the speech task? (1 = very little, 7 = very much)

**<Perceived Demand & Resources Regarding the Norm>**

Regarding the short speech you just completed, to what extent do you agree or disagree with the following statements? (1 = strongly disagree, 7 = strongly agree)

I’m not sure if other people will support my opinion on migrants.

It requires much effort to uphold my attitudes towards migrants in conversations with others.

I believe that other people hold the same attitudes towards migrants as I do.

I’m open to others’ opinions on migrants that are different from mine.

**<Perceived Demand & Resources Regarding Migration>**

The presence of migrants here brings about uncertainty regarding the future of the Netherlands.

The large number of migrants in the Netherlands endangers our economy and culture.

I feel prepared for the changes that migration will bring to the Netherlands.

I’m interested in multicultural experiences.

**<Perceived Threat>**

Regarding the relations between native Dutch and migrants in the Netherlands, to what extent do you agree or disagree with the following statements? (1 = strongly disagree, 7 = strongly agree)

Because of the arrival of migrants, Dutch people less quickly find a job.

It is more difficult for Dutch people to get a house due to migrants.

Migrants are a burden to the social welfare system in the Netherlands.

Migrants are a threat to the safety of Dutch people.

Because of the presence of migrants, the crime rate in the Netherlands increases.

Dutch identity is being threatened because there are too many migrants.

Dutch values and norms are undermined by the presence of migrants.

Moral and religious beliefs of Dutch people are being threatened because of the presence of migrants.

Migrants are a threat to the Dutch culture.

**<Social Distance>**

I would be happy to work with a migrant.

I would be happy to meet a migrant as a co-worker.

I would be happy to have a migrant marry into my family.

I would be happy to have a migrant as a close personal friend.

**<Feeling Thermometer>**

In general, on the following scale, how coldly or warmly do you feel towards migrants? (0 = very cold, 50 = neutral, 100 = very warm)

**<Emotions>**

Below is a list of different possible feelings or emotions. For each one, please indicate the extent to which you experience it in the context of the relations between native Dutch and migrants in the Netherlands. (1 = not at all, 7 = very much)

Fear of migrants

Anger towards migrants

Hatred for migrants

Contempt towards migrants

Anxiety about migrants

Sympathy for migrants

Guilt regarding the treatment of migrants

Hope for better relations between native Dutch and migrants

**<RWA & SDO>**

Now, show how much you agree or disagree with each idea below by selecting a number from 1 to 7 on the scale below. (1 = strongly disagree, 7 = strongly agree)

It’s great that many young people today are prepared to defy authority.

What our country needs most is discipline, with everyone following our leaders in unity.

God’s laws about abortion, pornography, and marriage must be strictly followed before it is too late.

There is nothing wrong with premarital sexual intercourse.

Our society does NOT need tougher government and stricter laws.

The facts on crime and the recent public disorders show we have to crack down harder on troublemakers, if we are going preserve law and order.

An ideal society requires some groups to be on top and others to be on the bottom.

Some groups of people are simply inferior to other groups.

No one group should dominate in society.

Groups at the bottom are just as deserving as groups at the top.

Group equality should not be our primary goal.

It is unjust to try to make groups equal.

We should do what we can to equalize conditions for different groups.

We should work to give all groups an equal chance to succeed.

**<Identification>**

Personally speaking, to what extent do you agree or disagree with the following statements? (1 = strongly disagree, 7 = strongly agree)

I feel strongly affiliated with the category of native Dutch.

Being a native Dutch is an important part of my identity.

I feel strongly committed to the native Dutch.

When I talk about native Dutch, I say “We” rather than “They”.

**Demographics**

Finally, here are some demographic questions.

**<Gender>**

What is your gender? (1 = female, 2 = male, 3 = non-binary, 4 = I prefer to identify myself:)

**<Age>**

When were you born?

**<Birth place>**

In which country were you born?

In which country was your mother born?

In which country was your father born?

**<Migration background>**

Do you identify yourself as one with or without a migration background?

With migration background

Without migration background

**<Ideology>**

On a left-right spectrum, how would you describe your political orientation? (1= very leftist, 4 = centrist, 7 = very rightist)

How social or liberal are your economic views? (1 = very social, 4 = neither social nor liberal, 7 = very liberal)

How progressive or conservative are your views on social issues? (1 = very progressive, 4 = neither progressive nor conservative, 7 = very conservative)

**<Migration group>**

When thinking of migrant groups, which is the first migrant group that comes into your mind?

Which are the other migrant groups that come into your mind?

**Appendix B. Analyses on Cardiovascular Measures During the Period of Reflection and Preparation for the Speech Task**

We computed HR, PEP, CO, and TPR reactivity scores, as well the TCI, for the speech preparation period. We then conducted the same analyses for the speech preparation period as we did for the speech task.

**Task Engagement**

A test for task engagement showed that HR reactivity (*M* = 11.71, *SD* = 7.90) was significantly greater than zero (i.e., baseline), *t*(201) = 21.08, *p* < .001, *d* = 1.48, and PEP reactivity (*M* = –3.68, *SD* = 10.34) was significantly smaller than zero, *t*(201) = 5.06, *p* < .001, *d* = 0.36, showing sufficient overall task engagement and goal relevance.

**Cardiovascular Measures of Threat and Challenge**

We first examined the main effect of the manipulation of changes in public opinion on each cardiovascular measure using one-way ANOVA. Additionally, we further tested the moderating role of political ideology using regression analysis, including the manipulation, ideology, and their interaction in the model. The manipulation of changes in public opinion was dummy-coded with the stability condition as the reference category.

The manipulation of changes in public opinion did not yield significant main effects on TCI, *F*(2, 199) = 0.61, *p* = .543, $\eta_{p}^{2}$ = .01. Furthermore, the regression analysis on TCI revealed no significant interaction between progressive change (vs. stability) and ideology, *b =* –0.42, *SE* = 0.36, *t*(194) = –1.19, *p* = .237, 95% CI [–1.13, 0.28], nor between conservative change (vs. stability) and ideology, *b =* –0.11, *SE* = 0.35, *t*(194) = –0.30, *p* = .762, 95% CI [–0.80, 0.58].

We then performed parallel analysis on the separate components of the TCI, namely CO and TPR. First, the ANOVA showed that the manipulation of changes in public opinion had no significant main effect on CO, *F*(2, 199) = 0.38, *p* = .681, $\eta_{p}^{2}$ = .00. The regression analysis on CO suggested no significant interaction between progressive change (vs. stability) and ideology, *b =* –0.11, *SE* = 0.10, *t*(194) = –1.05, *p* = .294, 95% CI [–0.31, –0.09], nor between conservative change (vs. stability) and ideology, *b =* –0.11, *SE* = 0.10, *t*(194) = –1.04, *p* = .299, 95% CI [–0.31, –0.09].

Likewise, the ANOVA showed that the manipulation of changes in public opinion had no significant main effect on TPR, *F*(2, 199) = 1.12, *p* = .328, $\eta_{p}^{2}$ = .01. Moreover, the regression analysis on TPR revealed no significant interaction between progressive change (vs. stability) and ideology, *b =* 98.28, *SE* = 94.57, *t*(194) = 1.04, *p* = .300, 95% CI [–88.24, 284.80], nor between conservative change (vs. stability) and ideology, *b =* –47.49, *SE* = 92.78, *t*(194) = –0.51, *p* = .609, 95% CI [–230.47, 135.49].

**Appendix C. Analyses with Economic and Social Ideologies as Separate Moderators**

We measured political ideology using three items that tapped into its general, economic, and social dimensions. General ideology showed a strong correlation with economic ideology, *r* = .46, *p* < .001, and social ideology, *r* = .52, *p* < .001. However, the correlation between economic and social ideologies, while significant, was relatively modest, *r* = .19, *p* = .007. This suggests that participants could identify as economically leftist but socially rightist, or vice versa. Providing this, we conducted regression analyses similar to those in the main text but included economic ideology and social ideology as moderators in separate models. Given that participants’ social ideology was much more skewed than economic ideology, we explored the simple slope effects for significant interactions at moderator levels 2 (leftist) and 6 (rightist) to enhance the comparability between analyses involving economic and social ideologies.

**The Moderating Role of Economic Ideology**

We conducted regression analysis on each outcome variable. The two dummy-coded variables for the manipulation of public opinion change (with stability condition as the reference category), economic ideology, and dummy × economic ideology interactions were included in the model.

***Cardiovascular Measures of Threat and Challenge***

The regression analysis on TCI suggested a significant interaction between progressive change (vs. stability) and economic ideology, *b =* –0.77, *SE* = 0.27, *t*(194) = –2.82, *p* = .005, 95% CI [–1.31, –0.23]. Specifically, a progressive change (vs. stability) in public opinion (vs. stability) led to higher challenge (lower threat) for economic leftists, *b =* 1.70, *SE* = 0.60, *t*(194) = 2.85, *p* = .005, 95% CI [0.52, 2.88], and higher threat (lower challenge) for economic rightists, *b =* –1.37, *SE* = 0.65, *t*(194) = –2.10, *p* = .037, 95% CI [–2.66, –0.09]. The interaction effect of conservative change (vs. stability) and economic ideology on TCI was not significant, *b =* –0.14, *SE* = 0.29, *t*(194) = –0.47, *p* = .636, 95% CI [–0.71, 0.43].

We then performed parallel analysis on the separate components of the TCI, namely CO and TPR. The interaction between progressive change (vs. stability) and economic ideology on CO was significant, *b =* –0.32, *SE* = 0.11, *t*(194) = –3.01, *p* = .003, 95% CI [–0.53, –0.11]. Decomposition of the interaction indicated that a progressive change (vs. stability) in public opinion increased CO (indicative of challenge) among economic leftists, *b =* 0.66, *SE* = 0.23, *t*(194) = 2.83, *p* = .005, 95% CI [0.20, 1.12], and decreased CO (indicative of threat) among economic rightists, *b =* –0.62, *SE* = 0.25, *t*(194) = –2.44, *p* = .016, 95% CI [–1.12, –0.12]. In contrast, there was no significant interaction between the conservative change (vs. stability) manipulation and economic ideology on CO, *b =* –0.08, *SE* = 0.11, *t*(194) = –0.73, *p* = .466, 95% CI [–0.30, 0.14].

Likewise, the regression analysis on TPR revealed a significant interaction between progressive change (vs. stability) and economic ideology, *b =* 183.77, *SE* = 91.06, *t*(194) = 2.02, *p* = .045, 95% CI [4.18, 363.37]. Decomposition of the interaction showed that a progressive change (vs. stability) in public opinion decreased TPR (indicative of challenge) among economic leftists, *b =* –448.21, *SE* = 199.43, *t*(194) = –2.25, *p* = .026, 95% CI [–841.54, –54.88], but not influenced it among economic rightists, *b =* 286.88, *SE* = 217.45, *t*(194) = 1.32, *p* = .189, 95% CI [–141.99, 715.75]. Moreover, the results indicated no significant interaction between the conservative change (vs. stability) manipulation and economic ideology on TPR, *b =* 11.24, *SE* = 96.28, *t*(194) = 0.12, *p* = .907, 95% CI [–178.64, 201.12].

***Self-report Measures***

The regression analysis on resource appraisal revealed no significant interaction between progressive change (vs. stability) and economic ideology, *b =* –0.15, *SE* = 0.18, *t*(195) = –0.82, *p* = .412, 95% CI [–0.50, 0.21], nor between conservative change (vs. stability) and economic ideology, *b =* –0.19, *SE* = 0.19, *t*(195) = –1.02, *p* = .311, 95% CI [–0.55, 0.18]. Likewise, neither the interaction effect of progressive change (vs. stability) and economic ideology on demand appraisals, *b =* 0.03, *SE* = 0.21, *t*(195) = 0.12, *p* = .902, 95% CI [–0.39, 0.44], nor that of conservative change (vs. stability) and economic ideology on demand appraisals, *b =* –0.04, *SE* = 0.22, *t*(195) = –0.16, *p* = .870, 95% CI [–0.47, 0.40], reached significance.

The same test for prejudice towards migrants as the outcome variable demonstrated a significant interaction between progressive change (vs. stability) and economic ideology, *b =* 5.56, *SE* = 2.46, *t*(195) = 2.26, *p* = .025, 95% CI [0.70, 10.42]. We probed into the simple slope effects, finding that a progressive change (vs. stability) in public opinion slightly decreased economic leftists’, *b =* –10.05, *SE* = 5.39, *t*(195) = –1.86, *p* = .064, 95% CI [–20.69, 0.58]—but increased economic rightists’, *b =* 12.19, *SE* = 5.88, *t*(195) = 2.07, *p* = .040, 95% CI [0.59, 23.78]—prejudice towards migrants. In contrast, there was no significant interaction between conservative change (vs. stability) and economic ideology on prejudice towards migrants, *b =* 3.76, *SE* = 2.55, *t*(194) = 1.48, *p* = .142, 95% CI [–1.27, 8.79].

**The Moderating Role of Social Ideology**

We conducted regression analysis on each outcome variable. The two dummy-coded variables for the manipulation of public opinion change (with stability condition as the reference category), social ideology, and dummy × social ideology interactions were included in the model.

***Cardiovascular Measures of Threat and Challenge***

The regression analysis on TCI suggested a significant interaction between conservative change (vs. stability) and social ideology, *b =* –0.51, *SE* = 0.25, *t*(194) = –2.02, *p* = .045, 95% CI [–1.00, –0.01]. Specifically, a conservative change (vs. stability) in public opinion (vs. stability) led to higher challenge (lower threat) for social leftists, *b =* 0.88, *SE* = 0.37, *t*(194) = 2.38, *p* = .018, 95% CI [0.15, 1.60], but did not have a significant influence for social rightists, *b =* –1.15, *SE* = 0.87, *t*(194) = –1.32, *p* = .188, 95% CI [–2.86, 0.59]. The interaction effect of progressive change (vs. stability) and social ideology on TCI was not significant, *b =* –0.41, *SE* = 0.26, *t*(194) = –1.58, *p* = .116, 95% CI [–0.92, 0.10].

However, the interaction between progressive change (vs. stability) and social ideology on CO was not significant, *b =* –0.11, *SE* = 0.10, *t*(194) = –1.09, *p* = .277, 95% CI [–0.31, –0.09], and nor was the interaction between conservative change (vs. stability) and social ideology on CO, *b =* –0.19, *SE* = 0.10, *t*(194) = –1.95, *p* = .053, 95% CI [–0.38, 0.00]. Regarding TPR, it was qualified by neither the interaction between progressive change (vs. stability) and social ideology, *b =* 148.95, *SE* = 85.51, *t*(194) = 1.74, *p* = .083, 95% CI [–19.69, 317.59], nor that between conservative change (vs. stability) and social ideology, *b =* 136.83, *SE* = 82.57, *t*(194) = 1.66, *p* = .099, 95% CI [–26.03, 299.68].

***Self-report Measures***

The regression analysis on resource appraisal revealed no significant interaction between progressive change (vs. stability) and social ideology, *b =* –0.22, *SE* = 0.17, *t*(195) = –1.32, *p* = .188, 95% CI [–0.55, 0.11], nor between conservative change (vs. stability) and social ideology, *b =* –0.21, *SE* = 0.16, *t*(195) = –1.34, *p* = .181, 95% CI [–0.53, 0.10]. Likewise, neither the interaction effect of progressive change (vs. stability) and social ideology on demand appraisals, *b =* –0.07, *SE* = 0.20, *t*(195) = –0.37, *p* = .715, 95% CI [–0.46, 0.32], nor that of conservative change (vs. stability) and social ideology on demand appraisals, *b =* –0.07, *SE* = 0.19, *t*(195) = –0.37, *p* = .710, 95% CI [–0.45, 0.30], reached significance.

The same test for prejudice towards migrants as the outcome variable demonstrated that the interaction between progressive change (vs. stability) and social ideology, *b =* 4.37, *SE* = 2.20, *t*(195) = 1.99, *p* = .048, 95% CI [0.04, 8.71], and that between conservative change (vs. stability) and social ideology, *b =* 5.32, *SE* = 2.11, *t*(195) = 2.52, *p* = .012, 95% CI [1.16, 9.48], were both significant. Decomposing the interactions, we found that a progressive change (vs. stability) in public opinion had a trend to increase social rightists’ prejudice towards migrants, *b =* 14.20, *SE* = 7.49, *t*(195) = 1.90, *p* = .059, 95% CI [–0.56, 28.97], but did not have such a influence among social leftists, *b =* –3.30, *SE* = 3.16, *t*(195) = –1.04, *p* = .299, 95% CI [–9.53, 2.94]. Moreover, a conservative change (vs. stability) in public opinion increased social rightists’ prejudice towards migrants, *b =* 18.11, *SE* = 7.33, *t*(195) = 2.47, *p* = .014, 95% CI [3.66, 32.56], but did not influenced social leftists’ prejudice, *b =* –3.18, *SE* = 3.08, *t*(195) = –1.03, *p* = .304, 95% CI [–9.25, 2.90].

**Appendix D. Analyses on Cardiovascular Measures Controlling for the Length of the Speech Task**

Given the variability in the length of the speech task, we conducted the same analyses on cardiovascular measures as in the main text but controlled for speech duration. The results are as follows.

On the TCI, an ANCOVA with speech duration being controlled for showed that the manipulation of changes in public opinion showed no significant main effects, *F*(2, 196) = 1.08, *p* = .340, $\eta_{p}^{2}$ = .01. Additionally, the regression analysis with speech duration as a control variable suggested a significant interaction between progressive change (vs. stability) and ideology, *b =* –0.81, *SE* = 0.36, *t*(193) = –2.25, *p* = .025, 95% CI [–1.52, –0.10]. As predicted, a progressive change (vs. stability) in public opinion (vs. stability) led to higher challenge (lower threat) for ideological leftists (–1*SD*: *b =* 0.97, *SE* = 0.45, *t*(193) = 2.16, *p* = .032, 95% CI [0.08, 1.85]) and slightly higher threat (lower challenge) for ideological rightists (+2*SD*: *b =* –1.19, *SE* = 0.69, *t*(193) = –1.71, *p* = .088, 95% CI [–2.56, 0.18]; +1*SD*: *b =* –0.47, *SE* = 0.43, *t*(193) = –1.09, *p* = .278, 95% CI [–1.31, 0.38]). The interaction effect of conservative change (vs. stability) and ideology on TCI was not significant, *b =* –0.59, *SE* = 0.35, *t*(193) = –1.69, *p* = .092, 95% CI [–1.28, 0.10].

We then performed parallel analysis on the separate components of the TCI, namely CO and TPR. First, regarding CO, the ANCOVA showed that the manipulation of changes in public opinion had no significant main effect, *F*(2, 196) = 0.14, *p* = .869, $\eta_{p}^{2}$ = .00. The regression analysis on CO showed a significant interaction between progressive change (vs. stability) and ideology, *b =* –0.28, *SE* = 0.14, *t*(193) = –1.97, *p* = .050, 95% CI [–0.56, –0.00]. Decomposition of the interaction indicated that a progressive change (vs. stability) in public opinion slightly increased CO (indicative of challenge) among ideological leftists (–1*SD*: *b =* 0.30, *SE* = 0.18, *t*(193) = 1.72, *p* = .087, 95% CI [–0.04, 0.65]), but did not influence CO among ideological rightists (+2*SD*: *b =* –0.44, *SE* = 0.27, *t*(193) = –1.61, *p* = .109, 95% CI [–0.98, 0.10]; +1*SD*: *b =* –0.19, *SE* = 0.17, *t*(193) = –1.13, *p* = .260, 95% CI [–0.52, 0.14]). In contrast, there was no significant interaction between the conservative change (vs. stability) manipulation and ideology on CO, *b =* –0.24, *SE* = 0.14, *t*(193) = –1.73, *p* = .085, 95% CI [–0.51, 0.03].

Likewise, regarding TPR, the ANCOVA showed that the manipulation of changes in public opinion had no significant main effect, *F*(2, 196) = 2.38, *p* = .095, $\eta_{p}^{2}$ = .02. The regression analysis on TPR revealed a significant interaction between progressive change (vs. stability) and ideology, *b =* 242.91, *SE* = 119.30, *t*(193) = 2.04, *p* = .043, 95% CI [7.61, 478.22]. Decomposition of the interaction showed that a progressive change (vs. stability) in public opinion decreased TPR (indicative of challenge) among ideological leftists (–1*SD*: *b =* –315.74, *SE* = 149.02, *t*(193) = –2.12, *p* = .035, 95% CI [–609.65, –21.82]), but did not influence it among ideological rightists (+2*SD*: *b =* 332.11, *SE* = 230.79, *t*(193) = 1.44, *p* = .152, 95% CI [–123.09, 787.31]; +1*SD*: *b =* 114.87, *SE* = 142.43, *t*(193) = 0.81, *p* = .421, 95% CI [–166.05, 395.79]). Moreover, the results indicated no significant interaction between the conservative change (vs. stability) manipulation and ideology on TPR, *b =* 147.89, *SE* = 115.59, *t*(193) = 1.28, *p* = .202, 95% CI [–80.10, 375.88].

**Appendix E. Analyses on Cardiac Output and Total Peripheral Resistance**

Similar to the analysis of TCI in the main text, we conducted parallel analyses on the individual components of TCI, namely CO and TPR. Table E1 summarises the estimated marginal means and their standard errors.

The ANOVA showed that CO was not significantly affected by the manipulation of changes in public opinion, *F*(2, 199) = 0.21, *p* = .807, $\eta_{p}^{2}$ = .00. The regression analysis on CO, however, showed a significant interaction between progressive change (vs. stability) and ideology, *b =* –0.33, *SE* = 0.14, *t*(194) = –2.36, *p* = .019, 95% CI [–0.61, –0.05]. Decomposition of the interaction indicated that a progressive change (vs. stability) in public opinion increased CO (indicative of challenge) among ideological leftists (–1*SD*: *b =* 0.35, *SE* = 0.18, *t*(194) = 2.01, *p* = .046, 95% CI [0.01, 0.70]), and slightly decreased CO (indicative of threat) among ideological rightists (+2*SD*: *b =* –0.53, *SE* = 0.27, *t*(194) = –1.94, *p* = .053, 95% CI [–1.06, 0.01]; +1*SD*: *b =* –0.23, *SE* = 0.17, *t*(194) = –1.38, *p* = .169, 95% CI [–0.57, 0.10]). In contrast, there was no significant interaction between the conservative change (vs. stability) manipulation and ideology on CO, *b =* –0.26, *SE* = 0.14, *t*(194) = –1.89, *p* = .060, 95% CI [–0.53, 0.01].

Likewise, regarding TPR, the ANOVA showed that the manipulation of changes in public opinion had no significant effect on it, *F*(2, 199) = 2.41, *p* = .093, $\eta_{p}^{2}$ = .02. However, the regression analysis on TPR revealed a significant interaction between progressive change (vs. stability) and ideology, *b =* 281.53, *SE* = 118.16, *t*(194) = 2.38, *p* = .018, 95% CI [48.49, 514.56]. Decomposition of the interaction showed that a progressive change (vs. stability) in public opinion decreased TPR (indicative of challenge) among ideological leftists (–1*SD*: *b =* –352.76, *SE* = 148.55, *t*(194) = –2.37, *p* = .019, 95% CI [–645.75, –59.78]), and slightly increased it (indicative of threat) among ideological rightists (+2*SD*: *b =* 398.06, *SE* = 229.38, *t*(194) = 1.74, *p* = .084, 95% CI [–54.33, 850.45]; +1*SD*: *b =* 146.29, *SE* = 142.26, *t*(194) = 1.03, *p* = .305, 95% CI [–134.29, 426.88]). Moreover, the results indicated no significant interaction between the conservative change (vs. stability) manipulation and ideology on TPR, *b =* 165.25, *SE* = 115.91, *t*(194) = 1.43, *p* = .156, 95% CI [–63.36, 393.86].

**Table E1**

*The Threat–Challenge Index (TCI), Cardiac Output (CO,) and Total Peripheral Resistance (TPR) as a Function of the Interaction Between Experimental Condition and Ideology*

|  | Leftist (ideology: M – 1 SD) | | | Rightist (ideology: M + 2 SD) | | |
| --- | --- | --- | --- | --- | --- | --- |
|  | Progressive change | Conservative change | Stability | Progressive change | Conservative change | Stability |
| TCI | 0.19 (0.32) | 0.16 (0.29) | –0.91 (0.32) | –0.3 (0.46) | 0.47 (0.49) | 1.13 (0.51) |
| CO | 0.51 (0.13) | 0.45 (0.11) | 0.16 (0.12) | 0.36 (0.18) | 0.49 (0.19) | 0.89 (0.20) |
| TPR | –133.28 (105.64) | –164.03 (95.44) | 219.48 (104.44) | 26.13 (153.76) | –314.71 (161.75) | –371.92 (170.20) |

*Note.* Entries are estimated marginal means with standard errors in parentheses. CO is measured in L/m, and TPR is in dyn·s·cm^–5^.

**Appendix F. Bivariate Correlations**

**Table F1**

*Bivariate Correlations Between Continuous Variables*

|  | M | SD | 1 | 2 | 3 | 4 | 5 | 6 | 7 |
| --- | --- | --- | --- | --- | --- | --- | --- | --- | --- |
| 1. TCI | –.00 | 1.79 | — |  |  |  |  |  |  |
| 2. CO | 0.44 | 0.70 | .90*** | — |  |  |  |  |  |
| 3. TPR | –83.63 | 594.08 | –.90*** | –.60*** | — |  |  |  |  |
| 4. Demand appraisal | 4.22 | 1.35 | .17* | .14* | –.16* | — |  |  |  |
| 5. Resource appraisal | 3.31 | 1.18 | –.00 | –.02 | –.02 | –.14* | — |  |  |
| 6. Prejudice towards migrants | 32.54 | 16.29 | –.02 | –.03 | .01 | .14* | –.15* | — |  |
| 7. Political ideology | 3.32 | 0.90 | .10 | .08 | –.09 | –.00 | –.11 | .40*** | — |

*Note*. Entries are Pearson’s r. * *p* < .050; *** *p* < .001.
